# Interleukin‐33 is a Novel Immunosuppressor that Protects Cancer Cells from TIL Killing by a Macrophage‐Mediated Shedding Mechanism

**DOI:** 10.1002/advs.202101029

**Published:** 2021-09-05

**Authors:** Jing Wu, Ziqing Chen, Stina L. Wickström, Juan Gao, Xingkang He, Xu Jing, Jieyu Wu, Qiqiao Du, Muyi Yang, Yi Chen, Dingding Zhang, Xin Yin, Ziheng Guo, Lasse Jensen, Yunlong Yang, Wei Tao, Andreas Lundqvist, Rolf Kiessling, Yihai Cao

**Affiliations:** ^1^ Department of Microbiology Tumor and Cell Biology Karolinska Institute Stockholm 171 65 Sweden; ^2^ Department of Pharmacy The Second Hospital of Shandong University Jinan Shandong 250000 China; ^3^ Department of Oncology and Pathology Karolinska Institute Stockholm 171 77 Sweden; ^4^ Institute of Gastroenterology Zhejiang University Hangzhou 310016 China; ^5^ School of Medicine Sichuan Provincial People's Hospital University of Electronic Science and Technology of China Chengdu 611731 China; ^6^ Department of Pancreatic Surgery West China Hospital Sichuan University Chengdu Sichuan 610045 China; ^7^ Division of Cardiovascular Medicine Department of Medical and Health Sciences Linkoping University Sweden; ^8^ Department of Cellular and Genetic Medicine School of Basic Medical Sciences Fudan University Shanghai 200032 China; ^9^ Center for Nanomedicine and Department of Anesthesiology Brigham and Women's Hospital Harvard Medical School Boston MA 02115 USA; ^10^ Karolinska University Hospital Solna Stockholm 171 64 Sweden

**Keywords:** cancer cells, cytolytic T cells, interleukin‐33, metalloprotease, T‐cell receptors

## Abstract

Recognition of specific antigens expressed in cancer cells is the initial process of cytolytic T cell‐mediated cancer killing. However, this process can be affected by other non‐cancerous cellular components in the tumor microenvironment. Here, it is shown that interleukin‐33 (IL‐33)‐activated macrophages protect melanoma cells from tumor‐infiltrating lymphocyte‐mediated killing. Mechanistically, IL‐33 markedly upregulates metalloprotease 9 (MMP‐9) expression in macrophages, which acts as a sheddase to trim NKG2D, an activating receptor expressed on the surface of natural killer (NK) cells, CD8+ T cells, subsets of CD4+ T cells, iNKT cells, and *γδ* T cells. Further, MMP‐9 also cleaves the MHC class I molecule, cell surface antigen‐presenting complex molecules, expressed in melanoma cells. Consequently, IL‐33‐induced macrophage MMP‐9 robustly mitigates the tumor killing‐effect by T cells. Genetic and pharmacological loss‐of‐function of MMP‐9 sheddase restore T cell‐mediated cancer killing. Together, these data provide compelling in vitro and in vivo evidence showing novel mechanisms underlying the IL‐33‐macrophage‐MMP‐9 axis‐mediated immune tolerance against cancer cells. Targeting each of these signaling components, including IL‐33 and MMP‐9 provides a new therapeutic paradigm for improving anticancer efficacy by immune therapy.

## Graphical abstract

T cells execute specific killing effects on cancer cells through recognition of cell surface molecules expressed in both cells. This article shows that interleukin‐33‐stimulated macrophages produce MMP‐9 that eliminates cell surface molecules in immune cells and cancer cells. Through this mechanism, MMP‐9 debilitates the T cell‐medicated anticancer effects.

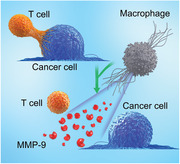

## 1 Introduction

In a solid tumor mass, a myriad of non‐cancerous cell types, including various immune cells, vascular cells, inflammatory cells, fibrotic cells, and even adipocytes, together with cancer cells coconstitute the tumor microenvironment (TME).^[^ ^1^ ^]^ These cancer and non‐cancer cells relentlessly cross‐communicate with each other and collectively determine tumor growth, progression, metastasis, and systemic responses in the cancer hosts. In some cancers such as pancreatic ductal adenocarcinoma, the tumor stromal component constitutes as much as over 90% of the total tumor mass and the stromal composition is reversely correlated with prognostic outcomes.^[^ ^2^ ^]^ Thus, drugs targeting the tumor stromal components such as antiangiogenic drugs and immunotherapeutics provide effective and comprehensive therapeutic modalities for treating various cancers.^[^ ^3^ ^]^ In fact, the tumor stroma‐based therapeutics have received the Food and Drug Administration‐approvals for treating various cancers in human patients.^[^ ^1b^ ^]^

Immunotherapy, including checkpoint inhibitors and cytolytic T cell‐based therapeutics, emerges as an effective therapeutic modality for treating solid and hematopoietic malignancies in human patients.^[^ ^4^ ^]^ In a small population of cancer patients, immunotherapy demonstrates a curative potential for treating various cancers such as melanoma, choriocarcinoma, and renal cancers.^[^ ^5^ ^]^ Adoptive T‐cell therapy (ACT), that is, cellular immunotherapy, employs isolation of T cells from the cancer hosts, expansion in their numbers in vitro, and sometimes genetic engineering for improving cancer cell recognition.^[^ ^6^ ^]^ Cellular immunotherapeutics, including tumor‐infiltrating lymphocytes (TILs); engineered T‐cell receptor (TCR); chimeric antigen receptor T cell (CAR‐T); and Natural Killer (NK) cell therapies, demonstrate imposing clinical efficacy for treating a broad‐spectrum of cancer types.^[^ ^7^ ^]^ For example, long‐term follow‐up of patients with metastatic melanoma demonstrates long‐lasting efficacy of tumor regression and curative potentials,^[^ ^8^ ^]^ also in patients who have failed on treatment with immune checkpoint inhibition.^[^ ^9^ ^]^ However, owing to genetic mutations and TME alterations, the therapeutic efficacy of ACT is generally modest and patients often acquire resistance.

Infiltration of inflammatory cells in tumors such as tumor‐associated macrophages (TAMs) is a hallmark of cancer.^2^, ^10^ ^]^ TAMs play imperious roles in cancer progression and metastasis by releasing tumor‐promoting growth factors and cytokines, producing proteases necessary for tumor invasion, hijacking cancer cells for metastasis, suppressing immune cell functions, stimulating angiogenesis and lymphangiogenesis, and cross‐communicating with cancer‐associated fibroblasts.^2^, ^10^, ^11^ ^]^ TAMs exhibit malleable phenotypic changes by expressing distinct cell surface markers and the M2‐TAM subpopulation correlates with an invasive phenotype for metastasis.^[^ ^11b^ ^]^ TAMs inhibit cancer immunosuppressive responses through several mechanisms:^[^ ^12^ ^]^ 1) production of immunosuppressive metabolites by depletion of amino acids; 2) expression of non‐canonical human leukocyte antigen (HLA) class I molecules such as HLA‐E and HLA‐G, which interact with inhibitory receptors expressed in various immune cells; 3) engagement of T cell inhibitory and apoptotic receptors; 4) production of immunosuppressive cytokines such as IL‐10 and TGF‐*β*1; 5) expression of signal regulatory protein alpha that increases the expression of CD47, a cell surface immunosuppressive molecule in cancer cells; 6) producing Treg‐recruiting chemokines such as CCL2, CCL5, CCL20, and CCL22 and Tregs inhibits T cell and NK cell activity; and 7) producing immunosuppressive prostaglandin E2.

Interleukin‐33 (IL‐33) belongs to the IL‐1 family and is synthesized in a broad range of cells, including stromal fibroblasts, perivascular cells, cancer cells, endothelial cells, adipocytes, and epithelial cells.^[^ ^13^ ^]^ Tumor‐derived growth factors such as platelet‐derived growth factor (PDGF) can upregulate IL‐33 expression in stromal fibroblasts and perivascular cells.^[^ ^10^ ^]^ IL‐33 displays its biological functions by activation of ST2 receptor expressed in immune cells and macrophages.^[^ ^10^, ^13^, ^14^ ^]^ IL‐33 instigates the transition of macrophages from the M1‐to‐M2 subtypes, the latter promotes metastasis through the production of MMP‐9.^[^ ^10^ ^]^ Blocking each component of the IL‐33‐ST2‐MMP‐9 axis markedly inhibited cancer metastatic potentials in preclinical models.^[^ ^10^ ^]^

The present study describes a previously unprecedented mechanism underlying the macrophage‐mediated immunosuppression in recognizing cancer cells for execution. In both in vitro and in vivo experimental settings, IL‐33 acted as an immunosuppressive cytokine by activation of macrophages, which successively inhibited TIL‐mediated specific killing of human primary melanoma cells. MMP‐9 was identified as a soluble sheddase in deteriorating T cell‐melanoma cell recognition by shedding natural killer group 2, member D (NKG2D) expressed in TILs and MHC class I expressed in melanoma cells. Inhibition of IL‐33 and MMP‐9 restore the TIL‐mediated killing effects in melanoma cells. Together, these findings unravel a novel mechanism of the macrophage‐mediated immunosuppression and targeting the IL‐33‐macrophage‐MMP‐9 axis provides a new therapeutic paradigm for improving benefits of immunotherapeutics.

## 2 Results

### 2.1 Immunosuppression of Tumor‐Infiltrating Lymphocyte‐Mediated Melanoma Killing by the Interleukin‐33‐Primed Macrophages

**Figure 1:**
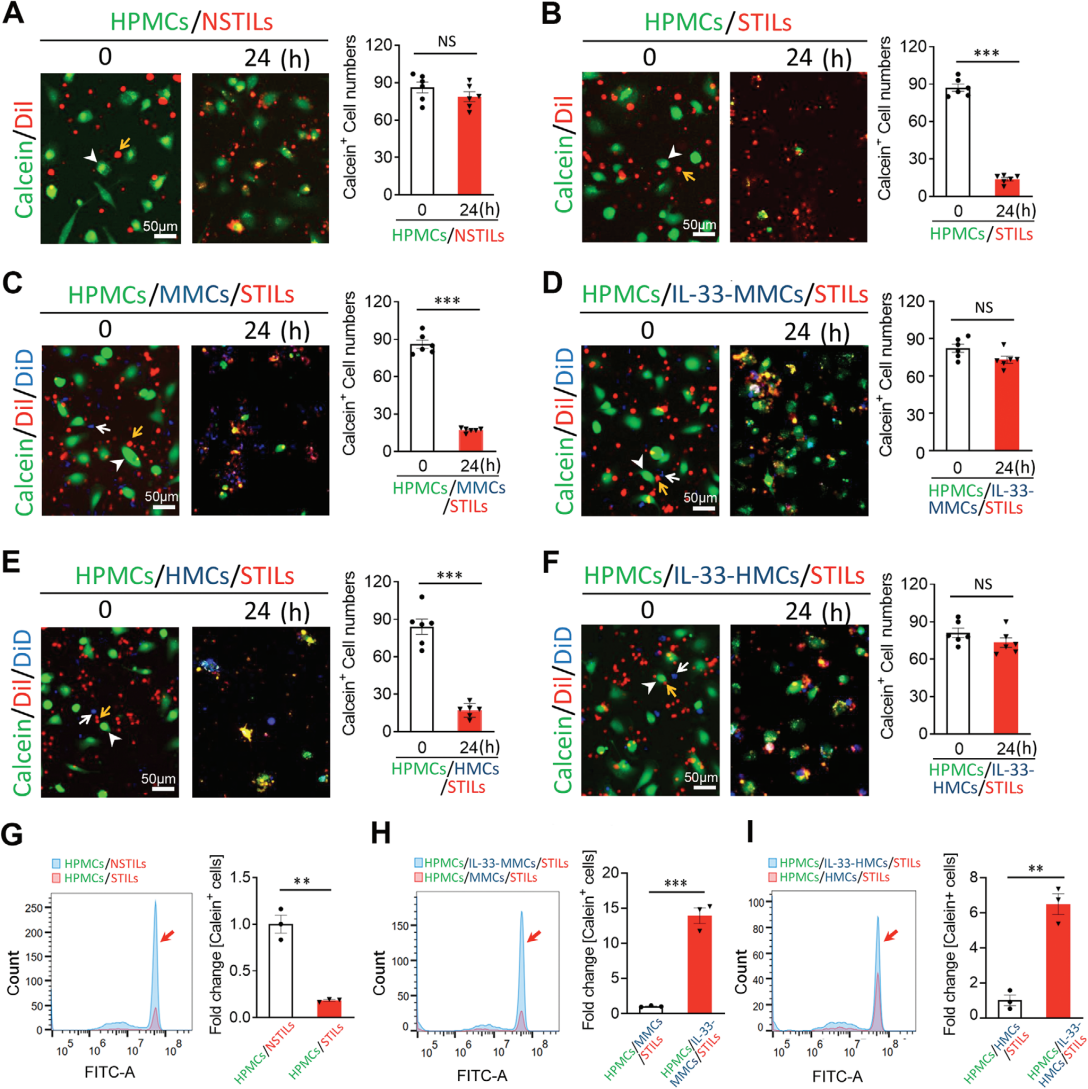
Debilitation of TIL‐mediated tumor killing by IL‐33‐primed macrophages. A–F) Randomized micrographs of calcein‐labeled HPMCs (green), DiI‐labeled NSTILs (red) or STILs (red), or plus DiD‐labeled macrophages (blue) were collected from each sample. Various combinations of cells were co‐incubated for 24 h. Yellow arrows point to TILs, white arrowheads indicate HPMCs, and white arrows point to macrophages. A) NTILs plus HPMCs (10:1). B) STILs plus HPMCs (10:1). C) Coculturing STILs, HPMCs, and MMCs (10:1:2). D) Coculturing STILs, HPMCs, and IL‐33‐stimulated MMCs (10:1:2). E) Coculturing STILs, HPMCs, and HMCs (10:1:2). F) Coculturing STILs, HPMCs, and IL‐33‐stimulated HMCs (10:1:2). Calcein‐positive HPMCs in all groups are quantified (*n* = 6 random fields per group, 10× magnification). Scale bar = 50 µm. G) FACS analysis of calcein‐positive HPMCs in samples containing NSTILs and STILs. H) FACS analysis of calcein‐positive HPMCs in samples containing STILs plus MMCs or IL‐33‐stimulated MMCs. I) FACS analysis of calcein‐positive HPMCs in samples containing STILs plus HMCs or IL‐33‐stimulated HMCs. Arrows in (G)–(I) indicate calcein positive cells. Data are mean determinants ± SEM; *n* = 3 samples per group. *p < 0.05; **p < 0.01; ***p < 0.001; NS, not significant, Unpaired Student's *t*‐test.

We have recently developed a two‐color system employing dual labeling of fluorescent calcein (green) in cancer cells and DiI in TILs (red).^[^ ^15^ ^]^ The non‐fluorescent calcein AM is a hydrophobic pro‐chemical molecule, which is effectively taken up by melanoma cells. Calcein AM is intracellularly converted into hydrophilic fluorescent calcein, which is released upon cell damage. Using this visualization system, the DiI‐labeled TILs and calcein‐labeled human primary melanoma cells (HPMCs) were cocultured in vitro to monitor the TIL‐executed killing effect. Coculture of HPMCs with non‐specific TILs (NSTILs) did not result in melanoma cell killing (**Figure** **1**A). By contrast, specific TILs (STILs) at the ratio 1:10 exhibited potent tumor killing effects after 24‐h coincubation with HPMCs (Figure 1B). A modest but significant killing effect was also observed at the ratio 1:5 between STILs and HPMCs (Figure S1A,B, Supporting Information).

To study the impact of macrophages on the TIL‐mediated cancer killing effect, mouse macrophages (MMCs) were co‐incubated with STILs and HPMCs. The naïve MMCs did not affect the HPMC killing effect by STILs (Figure 1C). However, the IL‐33‐stimulated MMCs completely protected HPMCs from STIL‐mediated killing (Figure 1D). To further validate the immunosuppressive effect of IL‐33, human macrophages (HMCs) were also pretreated with and without IL‐33. Similar with MMCs, the IL‐33‐primed HMCs fully incapacitated the STIL‐executed killing effects (Figure 1E,F). In concordant with these findings, FACS analysis produced nearly identical data showing the compromised killing effects of STILS in the presence of the IL‐33‐stimulated MMCs and HMCs (Figure 1G–I). On the basis of these findings, we conclude that IL‐33 is an immunosuppressive cytokine made by a macrophage‐mediated mechanism.

We next performed FACS analysis to quantitatively measure the proportion of cellular apoptosis and necrosis of HPMCs in the STIL‐HPMC coculture system. Annexin V and 7‐Amino‐Actinomycin D (7AAD) were used to define necrotic and apoptotic populations. The annexin V and 7AAD double positive population of necrotic HPMCs was significantly decreased in IL‐33‐MMCCM‐treated samples (Figure S1A,B, Supporting Information). Consistently, IL‐33 improved survivals of HPMCs in the STIL‐HPMC coculture system. Annexin V alone positive apoptosis also significantly reduced in the IL‐33‐treated coculture system (Figure S1A,B, Supporting Information).

### 2.2 A Soluble Factor Mediates the Interleukin‐33‐Macrophage‐Induced Immunosuppression

**Figure 2:**
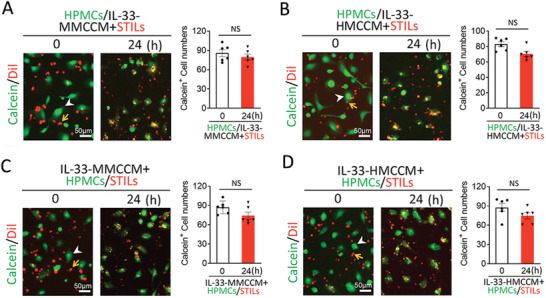
Soluble fraction of IL‐33‐stimulated macrophage mediates immunosuppression. A–D) Randomized micrographs of calcein‐labeled HPMCs (green) and DiI‐labeled STILs (red) that were non‐treated or treated with conditioned media derived from the IL‐33‐stimulated macrophages. Various combinations of cells were co‐incubated for 24 h. Yellow arrows point to STILs and white arrowheads indicate HPMCs. A) STILs were treated with IL‐33‐MMCCM for 24 h plus HPMCs (10:1). B) STILs were treated with IL‐33‐HMCCM for 24 h plus HPMCs (10:1). C) Coculturing STILs and HPMCs were treated with IL‐33‐MMCCM for 24 h (10:1). D) Coculturing STILs and HPMCs treated with IL‐33‐HMCCM for 24 h (10:1). Calcein‐positive HPMCs in all groups are quantified (*n* = 6 random fields per group, 10× magnification). Scale bar = 50 µm. Data are mean determinants ± SEM; *p < 0.05; **p < 0.01; ***p < 0.001; NS, not significant, Unpaired Student's *t*‐test.

To define the molecular identity of the IL‐33‐macrophage‐induced immunosuppression, the conditioned media of the IL‐33‐stimulated macrophages were used in our system. Interestingly, pretreatment of STILs with the conditioned medium from the IL‐33‐stimulated MMCs completely ablated the melanoma killing effects of STILs (**Figure** **2**A). These findings suggest that secreted factors released by macrophages mediate the immunosuppression of IL‐33. In support of this view, similar results were validated using the IL‐33‐treated HMC conditioned medium (Figure 2B). On the basis of these data, the soluble immunosuppressive molecules primarily target STILs to incapacitate their killing effects. However, we could not exclude the possibility of immunosuppressive effects of macrophage‐released factors on melanoma cells.

To study the impact of these potential immunosuppressive factors released by the IL‐33‐stimulated macrophages on melanoma cells, the conditioned media of the IL‐33‐treated MMCs (IL‐33‐MMCCM) were preincubated with melanoma cells. Noticeably, IL‐33‐MMCCM‐treated HPMCs evaded resistance against the STIL‐executed killing effect (Figure 2C). Likewise, the conditioned media of the IL‐33‐treated HMCs produced nearly identically protective effects (Figure 2D). Similar protective results were also obtained when the conditioned media from the IL‐33‐treated MMCs and HMCs were simultaneously added to the STIL‐HPMC coculture system (Figure S1C–F, Supporting Information). These findings demonstrate that the IL‐33‐treated macrophages release soluble factors that act on both melanoma cells and TILs to display immunosuppressive effects.

### 2.3 MMP‐9 Mediates the Interleukin‐33‐Macrophage‐Instigated Immunosuppression

**Figure 3:**
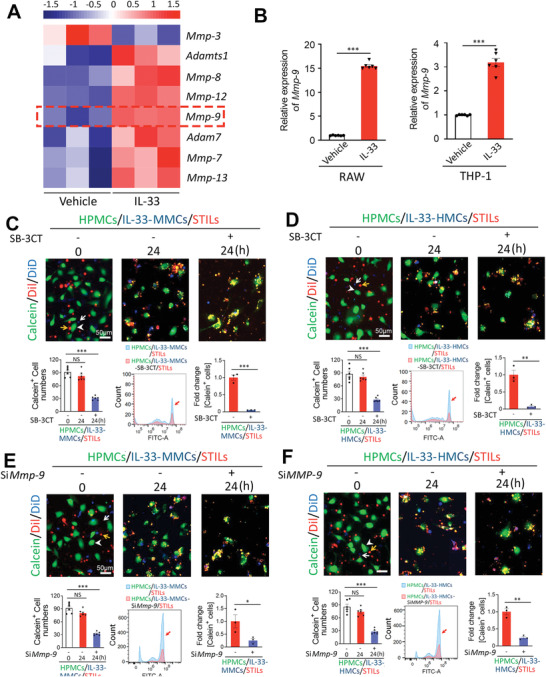
MMP‐9 mediates the IL‐33‐macrophage‐instigated immunosuppression. A) Heatmap of a myriad of MMPs genes by genome‐wide expression profiling of IL‐33‐MMCs (*n* = 3 samples per group). B) qPCR quantification of mouse *Mmp‐9* and human *MMP‐9* mRNA expression levels of IL‐33‐stimulated MMCs and HMCs (*n* = 6 samples per group). Randomized micrographs of calcein‐labeled HPMCs (green), DiI‐labeled STILs (red), or plus DiD‐labeled macrophages (blue) were collected from each sample. Various combinations of cells were co‐incubated for 24 h. C–F) Yellow arrows point to TILs, white arrowheads indicate HPMCs, and white arrows point to macrophages. C) Coculturing STILs, HPMCs, and IL‐33‐MMCs with or without SB‐3CT (10:1:2). D) Coculturing STILs, HPMCs, and IL‐33‐HMCs with or without SB‐3CT (10:1:2). E) Coculturing STILs, HPMCs, and IL‐33‐MMCs with or without si*Mmp‐9* (10:1:2). F) Coculturing STILs, HPMCs, and IL‐33‐HMCs with or without si*MMP‐9* (10:1:2). Calcein‐positive HPMCs in all groups are quantified (*n* = 6 random fields per group, 10× magnification). Scale bar = 50 µm. FACS analysis of calcein‐positive HPMCs in each group was also represented. Red arrows indicate calcein positive cells. Data are mean determinants ± SEM; *n* = 3 samples per group. *p < 0.05; **p < 0.01; ***p < 0.001; NS, not significant, Unpaired Student's *t*‐test.

Our recently published findings show that IL‐33 augments MMP‐9 expression in macrophages through the NF‐kB‐regulated transcription mechanism.^[^ ^10b^ ^]^ It was highly plausible that MMP‐9 could potentially act as the immunosuppressor released by the IL‐33‐stimulated macrophages. To explore this possibility, we employed both genetic and pharmacological approaches to block MMP‐9 functions. In consistent with previous findings, whole‐genome expression profiling demonstrated that a myriad of MMPs, including MMP‐9, MMP‐7, MMP‐8, MMP‐12, MMP‐13, and ADAM‐7, were markedly upregulated (**Figure** **3**A). qPCR analysis showed that MMP‐9 was expressed at the highest level in IL‐33‐treated macrophages.^[^ ^10b^ ^]^ Indeed, our present study validated that the elevated expression levels of the IL‐33‐treated MMCs and HMCs (Figure 3B). To inhibit MMP‐9 activity, an MMP‐9 inhibitor, SB‐3CT known to specifically block the MMP‐9 activity,^[^ ^10^, ^16^ ^]^ was used in our coculture system. In both MMCs and HMCs, addition of SB‐3CT completely restored the STIL‐triggered killing effects on HPMCs (Figure 3C,D). Similar to the pharmacologically inhibitory approach, genetic knockdown of MMP9 in MMCs and HMCs also restored the STIL‐mediated killing (Figure 3E,F). Addition of SB‐3CT to the conditioned media derived from IL‐33‐stimulated MMCs and HMCs also produced the restoration effects of STIL‐induced killing (Figure S2, Supporting Information). Similar to the pharmacological approach, genetic approach by knocking down MMP‐9 in MMCs and HMCs restored the T cell killing (Figure S3, Supporting Information). Together, these findings provide compelling evidence that MMP‐9 released by IL‐33‐stimulated macrophages mediates the immunosuppressive effects and protects melanoma cells from the STIL‐executed killing.

### 2.4 Interleukin‐33‐Stimulated Macrophages Protect Melanoma Cells from Tumor‐Infiltrating Lymphocyte Killing In Vivo

To validate our vitro findings, we chose a zebrafish tumor model for assessing the immunosuppressive effect of IL‐33‐stimulated macrophages. We have recently developed a zebrafish model to study the anticancer effect of cytolytic T cells.^[^ ^15^, ^17^ ^]^ Owing to the transparent and immune privilege features of zebrafish embryos, implantation of human or animal tumor cells into the perivitelline space (PVS) of zebrafish allows visualization of tumor growth, invasion, and metastasis.^[^ ^15^, ^17^ ^]^ Moreover, labeling each of the cellular components with miscellaneous colors allows investigators to study the role of each of cellular components in TME in tumor growth and metastasis. For example, the functions of the color‐labeled stromal macrophages and fibroblasts in cancer metastasis have been investigated in zebrafish and previously unprecedented new mechanisms have been proposed.^[^ ^15^, ^17^ ^]^

**Figure 4:**
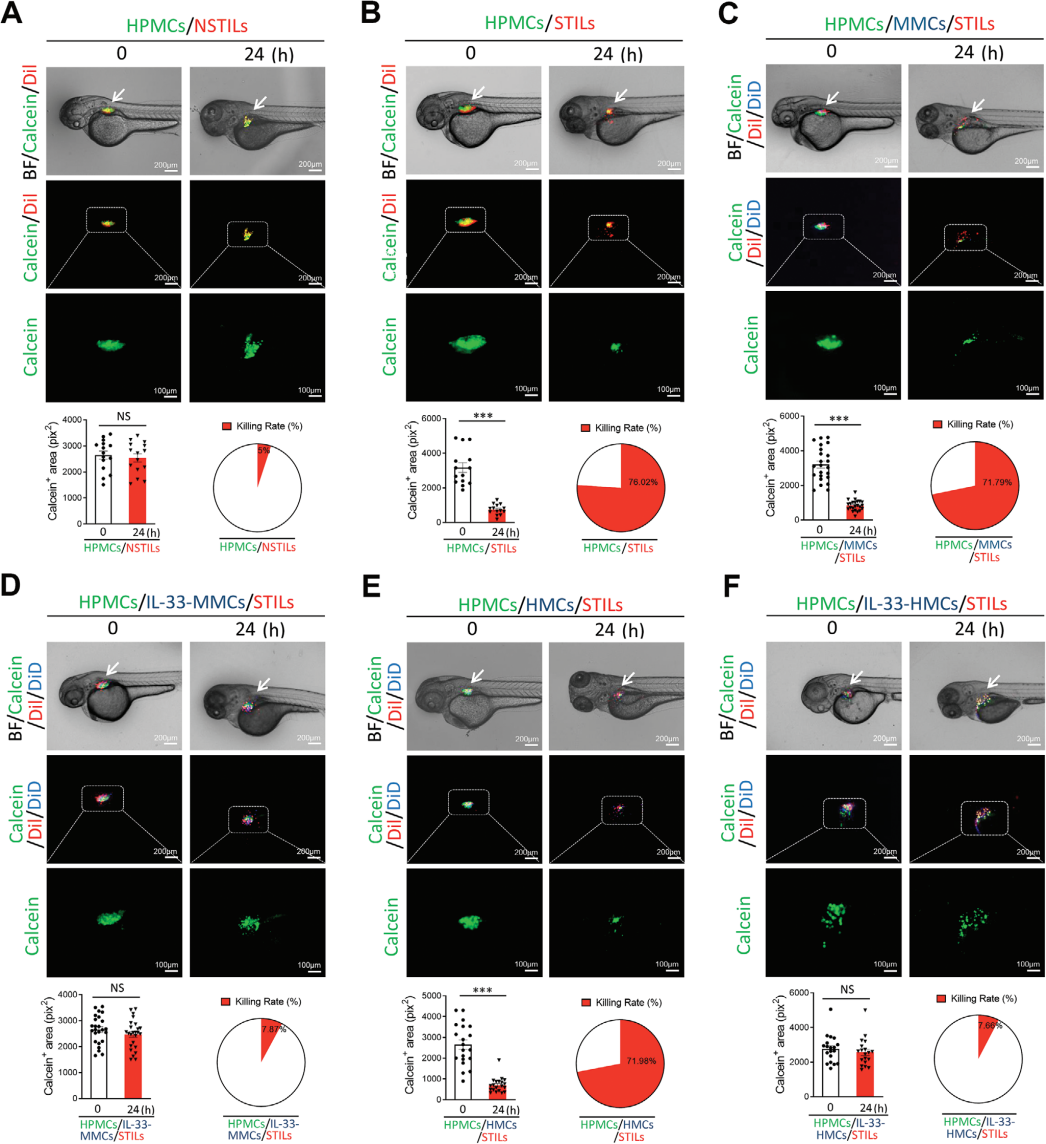
IL‐33‐stimulated macrophages protect HPMCs from TIL killing in zebrafish. A–F) Representative micrographs of calcein‐labeled HPMCs (green), DiI‐labeled TILs (red), or plus DiD‐labeled macrophages (blue) were collected at 0 and 24 h after co‐implantation into the zebrafish. White arrows point to injected cells. A) NTILs plus HPMCs (5:1). B) STILs plus HPMCs (5:1). C) Co‐injection of STILs, HPMCs, and MMCs (5:1:2). D) Co‐injection of STILs, HPMCs, and IL‐33‐stimulated MMCs (5:1:2). E) Co‐injection of STILs, HPMCs, and HMCs (5:1:2). F) Co‐injection of STILs, HPMCs, and IL‐33‐stimulated HMCs (5:1:2). Dashed lines rectangular and amplify the indicated regions. (Scale bars = 200 µm; amplified fields, 100 µm). Quantification of calcein‐positive areas in the zebrafish and killing rates of TILs were calculated (A, *n* = 15 samples per group; B, *n* = 14 samples per group; C, *n* = 23 samples per group; D, *n* = 25 samples per group; E, *n* = 20 samples per group; F, *n* = 19 samples per group). Data are mean determinants ± SEM; *p < 0.05; **p < 0.01; ***p < 0.001; NS, not significant, Unpaired Student's *t‐*test.

Using this in vivo model, the calcein‐labeled HPMCs, Dil‐labeled TILs, and DID‐labeled macrophages were implanted into the PVS of each zebrafish embryo. Implantation of human TILs, HPMCs, and HMCs did not cause toxicity (Figure S4A,B, Supporting Information). Co‐implantation of HPMCs and NSTILs did not result in immunological elimination of melanoma cells (**Figure** **4**A). Conversely, STILs exhibited potent tumor killing effect in the zebrafish body after 24‐h co‐implantation of HPMCs and STILs (Figure 4B). Triple implantation of HPMCs and STILs plus naïve MMCs or HMCs did not affect the melanoma killing activity by STILs (Figure 4C,E). By contrast, the IL‐33‐educated MMCs and HMCs completely protected cancer cells from the STIL‐mediated killing (Figure 4D,F). These data show that the IL‐33‐stimulated macrophages protect cancer cells from the STIL‐executed killing in vivo.

Similar to the in vitro coculturing system, STILs pretreated with the conditioned media from the IL‐33‐stimulated MMCs and HMCs demonstrated potent immunosuppressive effects in debilitating STIL‐mediated killing (Figure S4C,D, Supporting Information). Pretreatment of HPMCs with the conditioned media from the IL‐33‐stimulated MMCs and HMCs also abolished the STIL‐executed killing effects (Figure S4E,F, Supporting Information). These in vivo findings provide convincing evidence that the immunosuppressive effects of the IL‐33‐aumented macrophages are executed in both melanoma cells and TILs.

### 2.5 Inhibition of MMP‐9 Restores Tumor‐Infiltrating Lymphocyte‐Mediated Cancer Killing Effect In Vivo

**Figure 5:**
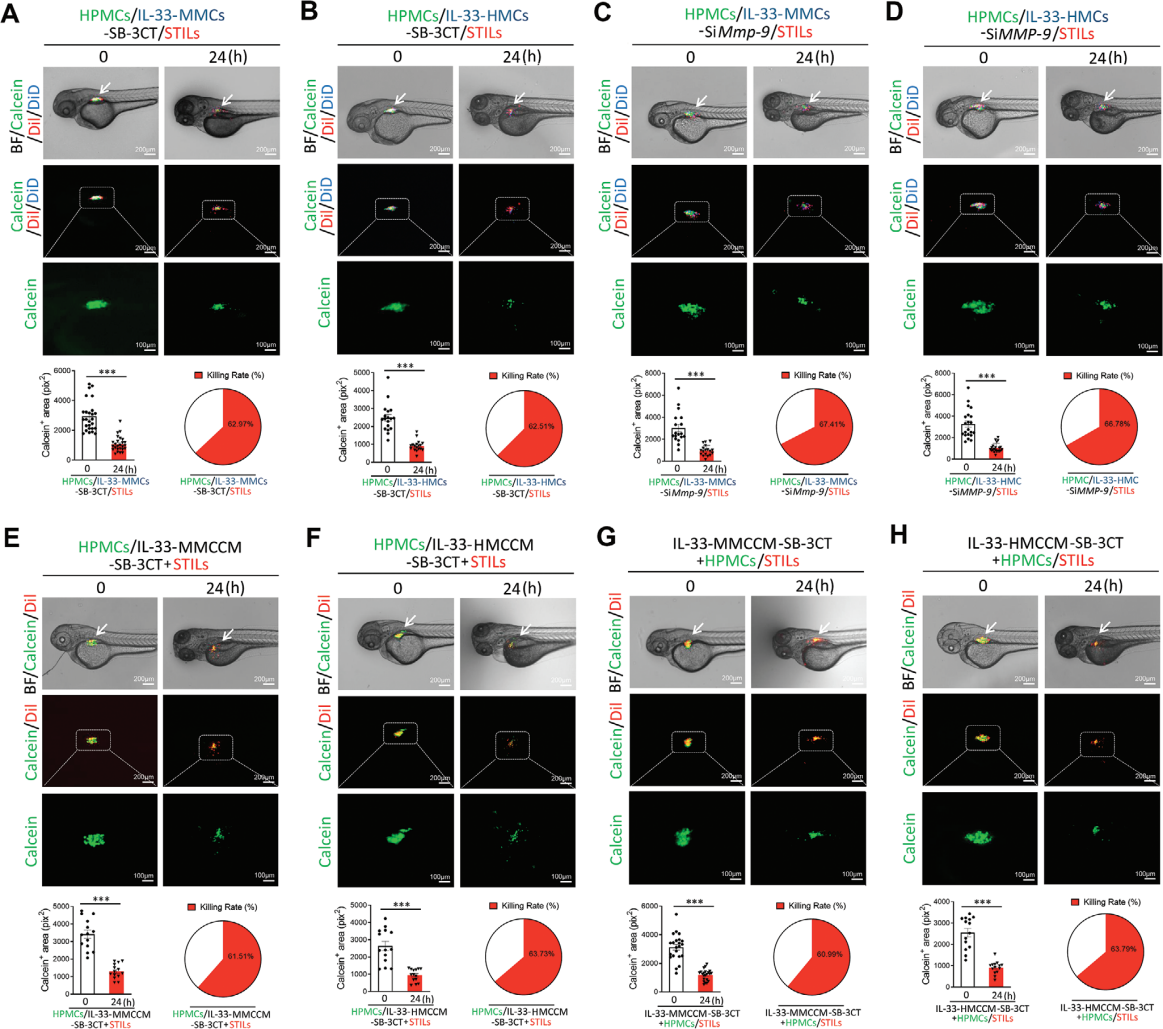
MMP‐9 inhibition restores TIL‐mediated cancer killing in vivo. Micrographs of zebrafish with HPMCs (green), IL‐33‐stimulated macrophages (blue) plus STILs (red) at 0 and 24 h post‐implantation. A–H) White arrows point to injected cells. A) Co‐injection of STILs, HPMCs, and IL‐33‐MMCs with SB‐3CT (5:1:2). B) Co‐injection of STILs, HPMCs, and IL‐33‐HMCs with SB‐3CT (5:1:2). C) Co‐injection of STILs, HPMCs, and IL‐33‐MMCs with si*Mmp‐9* (5:1:2). D) Co‐injection of STILs, HPMCs, and IL‐33‐HMCs with si*MMP‐9* (5:1:2). E) STILs treated with IL‐33‐MMCCM with SB‐3CT plus HPMCs (5:1). F) STILs treated with IL‐33‐HMCCM with SB‐3CT plus HPMCs (5:1). G) STILs plus HPMCs treated with IL‐33‐MMCCM with SB‐3CT (5:1). H) STILs plus HPMCs treated with IL‐33‐HMCCM with SB‐3CT (5:1). Quantification of calcein‐positive areas in the zebrafish and killing rates of TILs were calculated (A, *n* = 26 samples per group; B, *n* = 18 samples per group; C, *n* = 18 samples per group; D, *n* = 21 samples per group; E, *n* = 22 samples per group; F, *n* = 14 samples per group; G, *n* = 14 samples per group; H, *n* = 14 samples per group). Data are mean determinants ± SEM; *p < 0.05; **p < 0.01; ***p < 0.001; NS, not significant, Unpaired Student's *t‐*test.

Next, we investigated the impact of MMP‐9 inhibition on melanoma cell killing by TILs in vivo. Similar to the in vitro coculturing system, the SB‐3CT‐treated IL‐33‐educated MMCs and HMCs largely restored the STIL‐mediated killing effects on HPMCs (**Figure** **5**A,B). By the end of 24‐h incubation in the zebrafish body, the majority of calcein‐labeled HPMCs were eliminated by STILs and only minor populations of HPMCs remained. As SB‐3CT is defined as a specific inhibitor of MMP‐2/9, these data once again provide convincing evidence of the MMP‐mediated immunosuppression by IL‐33‐primed macrophages. Similarly, genetic knockdown of the mouse Mmp9 mRNA in MMCs and human MMP9 mRNA by their corresponding specific siRNAs produced nearly identical restoration effects of STIL‐mediated killing (Figure 5C,D). These genetic approaches of loss‐of‐function defined the MMP‐9 as a key mediator of immunosuppression by the IL‐33‐macrophage axis.

Pretreatment of STILs with conditioned media from the SB‐3CT‐treated IL‐33‐MMCs and IL‐33‐HMCs also rescued the tumor killing effects (Figure 5E,F). Consistent with the in vitro findings, pretreatment of HPMCs with conditioned media from the SB‐3CT‐treated IL‐33‐MMCs and IL‐33‐HMCs also restored the killing effects (Figure 5G,H). Similarly, knockdown of MMP‐9 by specific siRNAs in MMCs and HMCs restored the T cell killing (Figure S5, Supporting Information). Taken together, these in vivo experiments demonstrate that MMP‐9 is the key mediator of immunosuppression by IL‐33‐macrophages and it targets both STILs and melanoma cells.

### 2.6 MMP‐9 Impedes NKG2D in Tumor‐Infiltrating Lymphocytes and MICA/B in Cancer Cells

**Figure 6:**
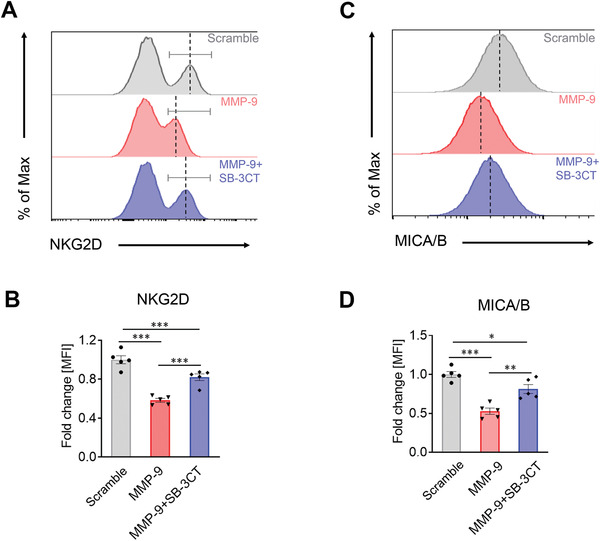
MMP‐9 cleaves NKG2D in TILs and MICA/B in HPMCs. Purified STILs were stimulated with rhMMP‐9 (5 µg mL^−1^) in the presence or absence of 20 µm SB‐3CT for 24 h. A) NKG2D expression of STILs were analyzed by FACS. B) Fold changes of flow cytometry mean fluorescence intensity (MFI) values were quantified. HPMCs were stimulated with rhMMP‐9 in the presence or absence of 20 µm SB‐3CT for 24 h. C) MICA/B expression levels in HPMCs were analyzed by FACS. D) Fold changes of flow cytometry MFI values were quantified. All experiments were repeated five times. Data are mean determinants ± SEM; *p < 0.05; **p < 0.01; ***p < 0.001 versus controls; NS, not significant, Unpaired Student's *t*‐test.

Knowing that both TILs and melanoma cells were the primary targets of the MMP‐9, we next devoted our efforts to define cell surface molecules that were affected by MMP‐9 in these cells. First, we performed FACS analysis of a panel of cell surface markers MMP‐9‐treated TILs associated with the TIL killing effects, including 1) CD3; 2) Fas ligand (FasL); 3) intercellular adhesion molecule 1 (also named CD54); and 4) IL‐2 receptor alpha (also named CD25) (Figure S6A–D, Supporting Information). These TIL cell surface markers remained unaltered after MMP‐9 treatment. Interestingly, the levels of NKG2D were significantly reduced after MMP‐9 treatment (**Figure** **6**A). NKG2D is a type 2 transmembrane protein expressed in NK cells and CD8^+^ T cells, which is concurrently required for activation of the TCR for recognition of the targeted cancer cells.^[^ ^18^ ^]^ SB‐3CT restored the NKG2D expression levels in MMP‐9‐treated TILs (Figure 6A,B). These findings demonstrate that NKG2D is an MMP‐9‐targeted protein expressed on the surface of TILs.

A similar FACS experimental approach was also applied to study the cell surface protein expressed in melanoma cells. MMP‐9 treatment of HPMCs markedly reduced the MICA/B expression levels (Figure 6C,D). MICA and MICB (MICA/B) are cell surface glycoproteins served as the NKG2D ligands. Inhibition of MMP‐9 by SB‐3CT largely restored the expression of MICA/B in HPMCs (Figure 6C,D). To generalize our findings, we studied the impact of MMP‐9 on MICA/B in the Hela cervical cancer that is known to express high levels of MICA/B.^[^ ^19^ ^]^ Similarly, MMP‐9 also decreased cell surface expression levels of MICA/B in Hela cells and SB‐3CT counteracted the MMP‐9 effect (Figure S6E,F, Supporting Information). Collectively, these findings provide insights into the mechanisms underlying the MMP‐9‐debilitated TILs killing effects.

### 2.7 Ablation of the Tumor‐Infiltrating Lymphocytes‐Human Primary Melanoma Cells Rosette Formation by the Interleukin‐33‐Stimulated Macrophages

**Figure 7:**
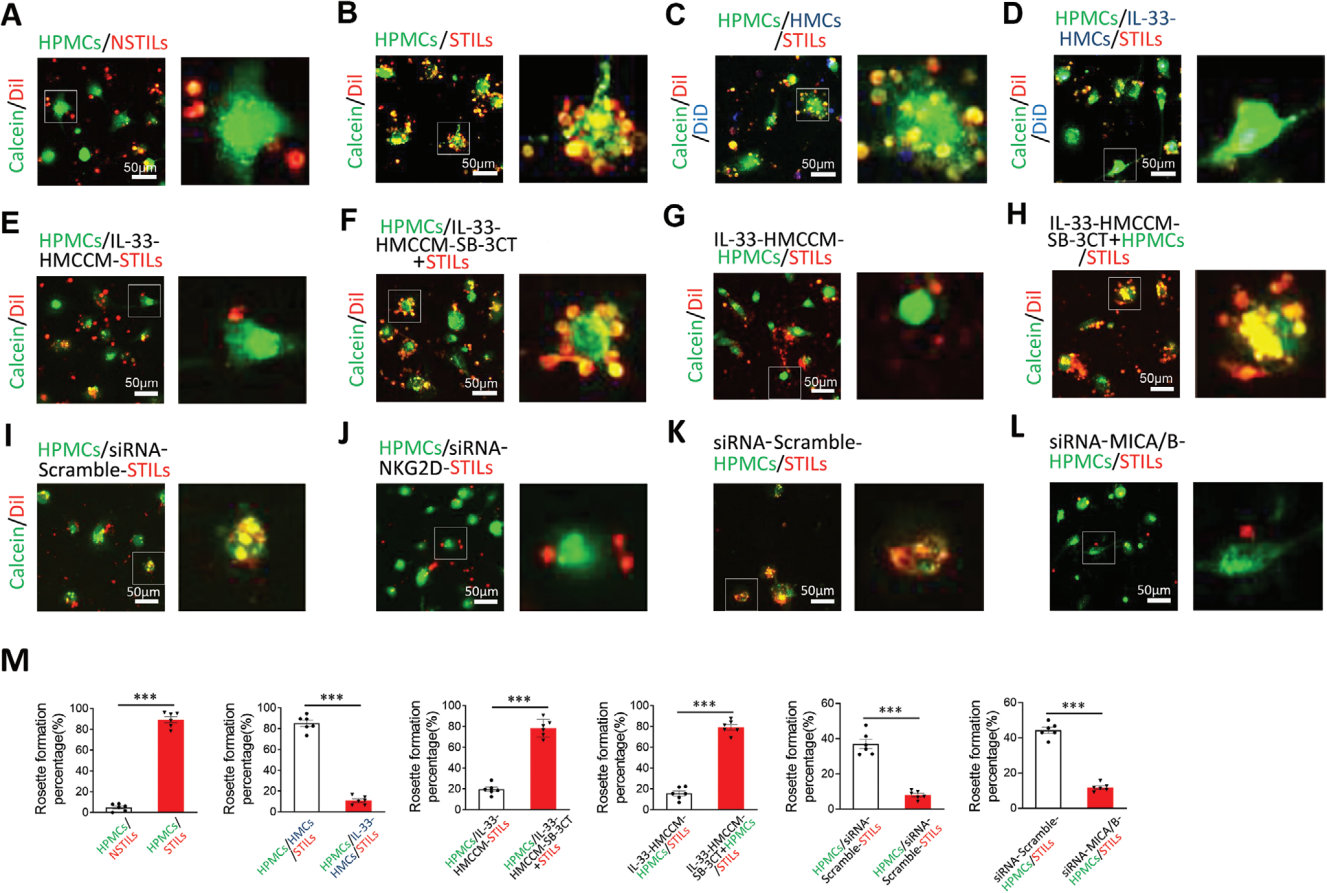
IL‐33‐stimulated macrophages ablate rosette formation between TILs and HPMCs. Randomized micrographs of calcein‐labeled HPMCs (green), DiI‐labeled NSTILs (red) or STILs (red), or plus DiD‐labeled macrophages (blue) were collected from each sample. Various combinations of cells were co‐incubated for 24 h. A) NTILs plus HPMCs (10:1). B) STILs plus HPMCs (10:1). C) Coculturing STILs, HPMCs, and HMCs (10:1:2). D) Coculturing STILs, HPMCs, and IL‐33‐stimulated HMCs (10:1:2). E) STILs treated with IL‐33‐HMCCM for 24 h plus HPMCs (10:1). F) STILs treated with IL‐33‐HMCCM containing 20 µM SB‐3CT plus HPMCs (10:1). G) Coculturing STILs and HPMCs treated with IL‐33‐HMCCM for 24 h (10:1). H) STILs plus HPMC treated with IL‐33‐HMCCM containing 20 µM SB‐3CT (10:1). I) Coculturing STILs with siRNA‐control and HPMCs for 24 h (10:1). J) Coculturing STILs with siRNA‐NKG2D and HPMCs for 24 h (10:1). K) Coculturing HPMCs with siRNA‐control and STILs for 24 h (10:1). L) Coculturing HPMCs with siRNA‐MICA/B and STILs for 24 h (10:1). M) The rosette formations were assessed and calculated (*n* = 6 random fields per group, 10× magnification). Scale bar = 50 µm. Dashed lines rectangular and amplify the indicated regions. Data are mean ± SEM; *p < 0.05; **p < 0.01; ***p < 0.001; NS, not significant, Unpaired Student's *t*‐test.

Reduction of NKG2D in TILs and MICA/B in HPMCs suggested to us that these cells might not be able to recognize each other. Recognition between TILs and targeted cancer cells is an initial and essential process necessary for TILs‐mediated killing. Morphologically, TILs recognize cancers by forming a rosette‐like structure in which multiple smaller T cells form a ring‐like structure around cancer cells. Indeed, in our experimental settings STILs and paired HPMCs formed the canonical rosettes, which were rarely found in NSTILs and HPMCs (**Figure** **7**A,B).

Co‐incubation of the STILs and HPMCs with HMCs did not significantly affect the rosette formation (Figure 7C). By contrast, the IL‐33‐stimulated HMCs markedly prevented the formation of STIL‐HPMC rosettes (Figure 7D). Similar inhibitory effects of rosette formation were also observed with the conditioned media from the IL‐33‐stimulated HMCs, but not from the controls (Figure 7E,F). Again, the MMP‐9 inhibitor SB‐3CT restored the STIL‐HPMC rosette formation (Figure 7G,H).

To further validate our findings, we took a genetic loss‐of‐function approach of knocking down NKG2D in TILs and MICA/B in HPMCs by their specific siRNAs. FACS analysis showed that siRNA‐NKG2D and a mixture of siRNA‐MICA and siRNA‐MICB effectively downregulated the expression levels of NKG2D in TILs and MICA/B in HPMCs (Figure S7, Supporting Information). Consistent with the knockdown levels, siRNA‐transfected cells showed impaired rosette formation (Figure 7I–M). These findings further support the role of NKG2D–MICA/B interactions in TIL and HPMC recognition. The rosette formation percentage was calculated as shown in Figure 7M. These findings validate the fact that MMP‐9 demolishes the recognition between TILs and melanoma cells by shedding their cell surface machineries, which are required for killing.

## 3 Discussion

Along with malignant progression, cellular compositions of the tumor stroma in TME relentlessly undergo alterations, which often elevate expression levels of signaling molecules that are beneficial for tumor growth, metastasis, and resistance to drug responses.^[^ ^1^, ^20^ ^]^ Understanding the interactive relations between various cell types and signaling pathways in TME provides a holistic view of the complex real‐life of the tumor mass and imperative clues for therapeutic intervention. Tumor cells often manipulate their microenvironment to evade immune surveillance and therapeutics by producing immunosuppressive signaling molecules. In this study, we provide compelling evidence to unravel a previously unprecedented mechanism by which inflammatory cells incapacitate the recognition between T cells and cancer cells and thereby deactivate the anticancer effect of immune cells. Using the matched‐pairs of primary human melanoma cells and TILs as examples, we demonstrate that the IL‐33‐primed macrophages acquire immunosuppressive ability to inactive TIL‐mediated killing. MMP‐9 was identified as a sheddase that mediates the immunosuppressive effect of macrophages. Because IL‐33 and MMP‐9 are often highly expressed in various tumors, this immunosuppressive mechanism most likely exists in other cancer types. Thus, targeting the IL‐33‐macrophage‐MMP‐9 signaling may provide a generalized therapeutic paradigm for treating a broad spectrum of cancer types.

**Figure 8:**
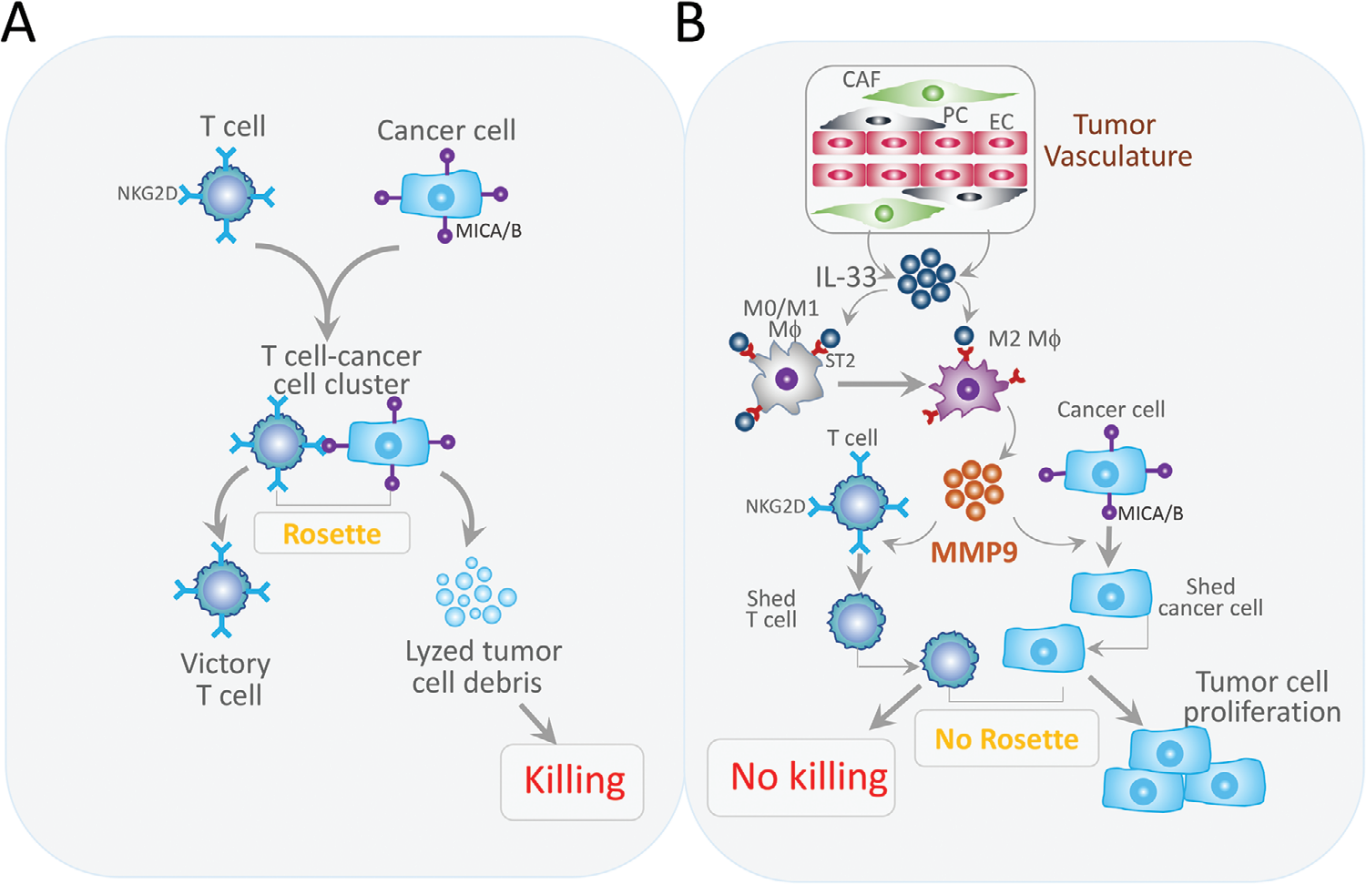
Mechanisms of IL‐33‐stimulated macrophages in protection of HPMCs from TIL‐executed killing. A) In solid tumors, T cells recognize cancer cells through interaction between specific receptor molecules expressed on the T cells such as TCR and NKG2D, and antigens such as MHC class I and MICA/B. T cells and the targeted cancers form rosette‐like flower structures, which permit specific cancer cell killing by T cells. B) In the tumor microenvironment, various stromal cellular components, including cancer‐associated fibroblasts (CAFs), tumor‐associated macrophages, and cells on the vessel wall coexist and undergo relentlessly changes. These stromal cellular components interact with each other to support tumor growth. For example, perivascular cells and CAFs produce high levels of IL‐33 in TME and IL‐33 stimulates the conversion of M1 macrophages to become the M2 type through the ST2 receptor expressed in macrophages. The IL‐33‐activated macrophages produce exceptionally high levels of MMP‐9. MMP9 acts as an immunosuppressive sheddase to cleave NKG2D on T cells and MICA/B on cancer cells. Ablation of NKG2D and MICA/B abolishes the formation of rosettes between T cells and cancer cells, thus debilitating the T cell‐mediated cancer killing effects.

Tumor cells propagate IL‐33 production in stromal cells by producing growth factors and cytokines such as PDGFs.^[^ ^10a^ ^]^ It appears that stromal fibroblasts and perivascular cells are the major cell types responsible for IL‐33 production in tumors.^[^ ^10^ ^]^ If so, IL‐33 links the vascular and fibrotic compartments to inflammatory cells in which ST2 receptor is expressed a variety of immune cells, including a large variety of immune cells, including T conventional cells, particularly type 2, regulatory T cells (Tregs), innate helper 2 cells (innate lymphoid cell type 2), M2 polarized macrophages, mast cells, eosinophils, basophils, neutrophils, NK, and iNKT cells.^[^ ^13b^ ^]^ These findings suggest that the IL‐33‐ST2 signaling may display immunosuppressive effects via activation of Tregs. Our present study provides an imperceptible mechanism of immunosuppressive function of IL‐33 through activation of macrophages. Stimulation of macrophages with IL‐33 markedly increases MMP‐9 production, which remodels the extracellular components to facilitate tumor invasion. MMP‐9 also acts as a sheddase to proteolytically trim cell surface proteins in both immune cells and cancer cells. In TILs, MMP‐9 significantly degrades NKG2D and abrogates its ability in recognizing cancer cell antigens (**Figure** **8**). Similarly, MMP‐9 also eliminates MICA/B expression in melanoma cells, evading T‐cell recognition for killing. Additive effects of immunosuppression from both TILs and melanoma cells would overwhelmingly impair their abilities for recognition and interaction. Although in this study we have provided compelling evidence of MMP‐9‐mediated immunosuppression, we cannot exclude the possibility of existence of similar mechanisms mediating other forms of MMPs in TME because IL‐33 induces a myriad of MMPs in macrophages.^[^ ^10b^ ^]^

On the basis of these findings, we propose that IL‐33 as an immunosuppressive cytokine in TME and targeting the IL‐33‐ST2 signaling would enhance the therapeutic efficacy of ACT‐based cellular immunotherapy. On the other hand, measurement of IL‐33 levels may provide a prognostic marker to predict therapeutic outcomes of cellular immune therapy. Development of neutralizing antibodies against IL‐33 or ST2 would provide an outstanding opportunity for effective therapy. Likewise, a soluble ST2 receptor may work equally well as a drug to neutralize IL‐33. It should be emphasized that these potential drugs may produce greater benefits in combination with immunotherapy than a single drug used alone.

In our initial findings, we discovered that the existence of a soluble immunosuppressive molecule released by the IL‐33‐stimulated macrophages and subsequent screening uncovers MMP‐9 as the potential inhibitor. Genetic knockdown and pharmacological inhibition of MMP‐9 nearly completely restore the TIL‐mediated killing. These findings are unexpectedly surprising because MMP‐9 is defined as a matrix protease. On the basis of these data, we speculate that simultaneously targeting MMP‐9 in combination with ACT would produce improved therapeutic benefits relative to ACT alone. Given the fact that MMP‐9 inhibitors alone lack clinical benefits for cancer therapy, these drugs may become an important therapeutic component in combination settings as shown in our study. This therapeutic paradigm warrants future validation in clinical settings.

In essence, our findings demonstrate that a complex regulation mechanism in TME controls anticancer immune response. Probably, the IL‐33‐macrophage‐MMP‐9 provides merely an example of immunosuppression and similar immunosuppressive signaling molecules likely coexist in TME. If MMP‐9 is defined as an immunosuppressive sheddase, other signaling molecules that induce MMP‐9 production would potentially inhibit anticancer effects of ACT. In addition to MMP‐9, other MMPs and ADAMs may also display similar shedding functions on T cells, cancerous cells, or both. Thus, simultaneously targeting these immunosuppressive molecules and proteases represents a new therapeutic paradigm to improve the therapeutic benefits of ACT.

## 4 Conclusion

IL‐33 operates the interactions between vascular/fibrotic cells and inflammatory macrophages to coordinately debilitate the cancer killing effect of cytolytic T cells by enhancing the proteolytic activity of MMPs. The IL‐33‐TAM‐MMP‐9 signaling is a novel immunosuppressive pathway by shedding antigen in cancer cells and antigen recognizing surface molecules in T cells (Figure 8). Targeting the IL‐33‐TAM‐MMP‐9 axis provides a novel therapeutic paradigm for treating various cancers by enhancing the effectiveness of immunotherapeutics.

## 5 Experimental Section

### Primary Cells and Cell Lines

Peripheral blood mononuclear cells (PBMC) were isolated from buffy coat obtained from healthy human volunteers (Karolinska University Hospital Blood Bank) using Ficoll gradient centrifugation (GE Healthcare) according to the manufacturer's instructions. Approval for collection of tumor material for production of TIL and primary melanoma cell lines was obtained from the Swedish Ethical Review Authority (Dnr 2020‐07099) and all patients provided written informed consent in accordance with the Declaration of Helsinki. STILs and NSTILs were expanded as previously described.^[^ ^21^ ^]^ HPMCs and RAW 264.7 cells were cultured in 10% FBS Dulbecco′s Modified Dulbecco Media (Thermo Fisher Scientific) containing 100 U mL^−1^ penicillin/streptomycin. THP‐1 cells were grown in RPMI‐1640 containing 10% FBS and 100 U mL^−1^ penicillin/streptomycin. All cells were negative for mycoplasma by routine tests using a Mycoplasma Detection Kit (Lonza Inc.).

### Drug Treatment

The mouse RAW 264.7 macrophages and human THP‐1 macrophages were starved by incubation overnight with 2% FBS, followed by stimulation with 50 ng mL^−1^ recombinant human IL‐33 (NOVUS, Cat. No. 3625‐IL‐010) for 24 h. In some experiments, SB‐3CT was added to macrophages at a final concentration of 20 µm and conditioned media from the drug‐treated and vehicle‐treated cells were collected after 24‐h treatment. Specific siRNAs targeting human MMP9 and mouse Mmp9 RNAs were used for knocking down MMP9 expression in THP‐1 and RAW 264.7 cells. After 48 h transfection, conditioned media were collected. rhMMP‐9 was added to STILs or HPMCs at a concentration of 5 µg mL^−1^ for 24 h for FACS detection.

### Zebrafish Tumor Model

All zebrafish experiments were performed according to the guideline by the Karolinska Institute Zebrafish Core Facility. Wild‐type AB strains of zebrafish were raised at 28 °C under standard conditions. At 24 h‐post‐fertilization (hpf), zebrafish embryos were transferred to an E3 medium containing 0.2 mmol L^−1^ 1‐phenyl‐2‐thio‐urea (Sigma) to prevent pigmentation. Embryos were anesthetized with 0.04 mg mL^−1^ of tricaine (MS‐222, Sigma) at 48 hpf prior to microinjection. HPMCs, TILs (both STILs and NSTILs), and macrophages (MMCs and HMCs) were labeled in vitro with 0.5 µm calcein‐AM (Invitrogen, C3100MP), 1 µg mL^−1^ of 1,1′‐dioctadecyl‐3,3,3′,3′‐tetramethylindocarbocyanine perchlorate (DiI, Sigma), and 2 mg mL^−1^ Vybrant DiD‐labeling solution (Life Technologies), respectively. Approximately 100–200 HPMCs or a mixture of 100–200 HPMCs and 200–400 macrophages were co‐injected or sequentially injected into the PVS of each embryo using an Eppendorf microinjector. Approximately, 500 STILs or NSTILs were injected into the same PVS location of each fish. After injection, zebrafish embryos were examined by a confocal microscope (Nikon Eclipse C1) and images were captured. The injected zebrafish embryos were further incubated at 33 °C for 24 h and analyzed by confocal microscopy. Calcein (green) positive areas were quantified to monitor the killing ability of TILs using ImageJ software.

### FACS Analysis

To assess the killing ability of TILs, calcein‐AM‐labeled HPMCs, pretreated HPMCs, and HPMCs mixed with non‐stimulated or IL‐33‐stimulated macrophages were seeded in a U‐bottom 96‐well plate. The ratio of tumor cells and macrophages were 1.0 × 10^4^ cells:2.0 × 10^4^ cells per well, respectively. Approximately, 1.0 × 10^5^ STILs, NSTILs and pretreated STILs were seeded in each well. After 24‐h incubation at 37 °C, the supernatants were discarded, cells were harvested, and stained by FACS. STILs and HPMCs were incubated for 24 h with 5 µg mL^−1^ rhMMP‐9 (Abcam, Cat. No. ab168863) or a mixture of rhMMP‐9 and SB‐3CT. STILs and HPMCs were washed by a buffer containing PBS and 2% FBS, followed by staining with an AQUA Live/Dead cell marker (Invitrogen, Cat. No. L34965), a PerCP/Cyanine5.5 anti‐human CD3 antibody (Biolegend, Cat. No. 317336), an APC anti‐human NKG2D antibody (Biolegend, Cat. No. 320808), an APC anti‐human MICA/B antibody (Biolegend Cat. No. 320907), a Pacific Blue anti‐human CD25 (Biolegend Cat. No. 302626), a PE anti‐human Fas‐L (Biolegend Cat. No. 306407), and a PE anti‐human CD54 (Biolegend Cat. No. 322708). After incubation with the antibody cocktail for 30 min, cells were detected by a Novocyte flow cytometer (ACEA biosciences). Annexin V‐FITC (Biolegend Cat. 640906) and 7AAD (Thermo fisher Cat. A1310) were used to define the necrosis and apoptosis populations. Cultured cells were washed with an Annexin‐binding buffer (Biolegend Cat. 422201) according to the manufacture´s instruction. The necrotic and apoptotic cells were detected by a Novocyte flow cytometer (ACEA biosciences). All the data were analyzed by Flowjo software (BD).

### Fluorescent Analysis

Calcein‐AM‐labeled HPMCs or pretreated HPMCs (5.0 × 10^4^) were seeded into a 12‐well plate. DiI‐labeled STILs, NSTILs, or pretreated STILs were added in triplicates into corresponding wells. A mixture of calcein‐AM‐labeled HPMCs and DiD‐labeled macrophages (1.0 × 10^5^) or IL‐33‐stimulated macrophages (1.0 × 10^5^) were seeded into a 12‐well plate. DiI‐labeled STILs (5.0 × 10^5^) with or without 20 µm SB‐3CT were added, followed by further incubation for 24 h. Images were captured randomly under a confocal microscope (Nikon Eclipse C1) and the data were analyzed by an ImageJ software.

### qPCR

A 2‐mercaptoethanol (Cat. No. 3148, Sigma)‐containing lysis buffer was used to lyze cells. Total RNA concentrations were measured by a Nanodrop apparatus (Thermo Scientific) and were reversely transcribed into cDNAs using a RevertAid cDNA synthesis kit according to the Manufacturers’ instructions (Cat. No. K1632, Thermo Scientific). qPCR was performed using a Power SYBR Green PCR Master Mix (Applied Biosystems) and a StepOnePlus detectable system (Applied Biosystems). Sequences of the paired primers were as follows: mouse‐Mmp‐9: forward, 5′‐GTCCAG ACC AAG GGGT ACA GC‐3′; reverse, 5′‐ATA CAG AGG GTA CAT GAG CG‐3′; mouse‐*β*‐actin; forward, 5′‐AGG CCC AGA GCA AGA GAG G‐3′; reverse, 5′‐TAC ATG GCT GGG GTG TTG AA‐3′. human‐mmp‐9; forward, 5′‐CAT CCG GCA CCT CTA TGG TC‐3′; reverse, 5′‐CAT CGT CCA CCG GAC TCA AA‐3′ and human‐*β*‐actin: forward, 5′‐ATT GCC GAC AGG ATG CAG AA‐3′; and reverse, 5′‐GCT GATCCA CAT CTGCTG GAA‐3′. The *β*‐actin was used as an internal control to normalize the amount and quantitative data were presented.

### RNA Silencing

An siRNA‐based approach was used to knockdown mouse Mmp‐9 mRNA in RAW 264.7 cells and human MMP‐9 in THP‐1 cells. Transfection was performed according to the manufacturer's instructions using the Bio‐Rad siLentFect lipid transfection kit (Bio‐Rad, Hercules, CA, USA). After 48 h, the cells were analyzed for expression levels of MMP‐9 using a qPCR method. The following siRNAs sequences were used: human‐MMP‐9, 5′‐GCGCUGGGCUUAGAUCAUUTT‐3′; mouse‐Mmp‐9, 5′‐GCACUGGGCUUAGAUCAUUTT‐3′, and control 5´‐UUCUCCGAACGUGUCACGUTT‐3′; For the knockdown of human‐NKG2D, human‐MICA, and human‐MICB, the authors used ON‐TARGET plus SMARTpool siRNAs from Horizon Discovery with catalog number as follows: L‐012391‐00‐0005, L‐187896‐00‐0005, and L‐012178‐00‐0005.

### Statistical Analysis

Data of mean determinants were presented as ±SEM. No statistical methods were used to predetermine sample sizes, and the exact values of *n* (sample size) are provided in each figure legends. In vitro studies: calcein positive cells detected in coculture experiments were calculated using ImageJ software; calcein positive cells detected in FACS were calculated using Flowjo software. In vivo studies: calcein positive areas in zebrafish were calculated using ImageJ software. Statistical analysis of data was performed using a standard Unpaired Student's *t*‐test by GraphPad Prism 8.0. *p < 0.05; **p < 0.01; ***p < 0.001; NS, not significant.

## Conflict of Interest

The authors declare no conflict of interest.

## Author Contributions

Y.C. generated the ideas and designed experiments. Jing.W. performed the most experiments. Z.C. and Jing.W. performed FACS analysis. S.L.W. isolated primary TILs, PBMCs, and HPMCs for this study. J.G., X.H., X.J., Jieyu.W., Q.D., M.Y., Y.C., D.Z., and X.Y. participated in experimentation. Z.G., L.J., Y.Y., W.T., A.L., and R.F. provided important materials and reagents. Y.C. wrote the manuscript.

## Supporting information

Supporting Information

## Data Availability

The data that support the findings of this study are available from the corresponding author upon reasonable request.
