# Supplementary material for: Interleukin‐33 is a Novel Immunosuppressor that Protects Cancer Cells from TIL Killing by a Macrophage‐Mediated Shedding Mechanism
Source: Adv Sci (Weinh). 2021 Sep 5;8(21):2101029. doi: 10.1002/advs.202101029 (PMC8564439; doi:10.1002/advs.202101029)
Supplement: Supplementary file 1 — Supporting Information [file ADVS-8-2101029-s001.pdf]

© 2021 Wiley-VCH GmbH

ADVANCED SCIENCE
Open Access

Supporting Information

for *Adv. Sci.,* DOI: 10.1002/advs.202001029

IL-33 is a novel immunosuppressor that protects cancer cells from TIL killing by a macrophage-mediated shedding mechanism

*Jing Wu[1,2], Ziqing Chen[3], Stina L Wickström[3], Juan Gao[1], Xingkang He[1,4], Xu Jing[1], Jieyu Wu[1], Qiqiao Du[1], Muyi Yang[3], Yi Chen[3], Dingding Zhang[1,5], Xin Yin[1], Ziheng Guo[6], Lasse Jensen[7], Yunlong Yang[8], Wei Tao[9], Andreas Lundqvist[3], Rolf Kiessling[3,10] and Yihai Cao[1]**

Supplementary Information

IL-33 is a novel immunosuppressor that protects cancer cells from TIL killing by a macrophage-mediated shedding mechanism

Jing Wu[1,2], Ziqing Chen[3], Stina L Wickström[3], Juan Gao[1], Xingkang He[1,4], Xu Jing[1], Jieyu Wu[1], Qiqiao Du[1], Muyi Yang[3], Yi Chen[3], Dingding Zhang[1,5], Xin Yin[1], Ziheng Guo[6], Lasse Jensen[7], Yunlong Yang[8], Wei Tao[9], Andreas Lundqvist[3], Rolf Kiessling[3,10] and Yihai Cao[1]*

[1]Department of Microbiology, Tumor and Cell Biology, Karolinska Institute, 171 65 Stockholm, Sweden

[2]Department of Pharmacy, The Second Hospital of Shandong University, Jinan, Shandong Province, China

[3]Department of Oncology and Pathology, Karolinska Institutet, 171 77 Stockholm, Sweden

[4]Insitute of Gatroenterology, Zhejiang University, Hangzhou, 310016, China

[5]School of Medicine,Sichuan Provincial People's Hospital, University of Electronic Science and Technology of China ， Chengdu ,611731,China

[6]Department of Pancreatic Surgery, West China Hospital, Sichuan University, Chengdu, China

[7]Division of Cardiovascular Medicine, Department of Medical and Health Sciences, Linkoping University, Sweden

[8]Department of Cellular and Genetic Medicine, School of Basic Medical Sciences, Fudan University, Shanghai, China

[9]Center for Nanomedicine and Department of Anesthesiology, Brigham and Women's Hospital, Harvard Medical School, Boston, MA, 02115, United States

[10] Karolinska University Hospital, 171 64 Solna, Stockholm, Sweden

Key words: Interleukin-33, cytolytic T cell, cancer cell, T cell receptor, metalloprotease

Running title: The IL-33-macrophage-MMP axis mitigates the cancer killing capacity of T cells

*Correspondence, galley proofs and reprint requests should be primarily addressed to: Yihai Cao, M.D., Ph.D., Department of Microbiology, Tumor and Cell Biology, Karolinska Institutet, 171 77 Stockholm, Sweden. Tel: (+46)-8-5248 7596, Fax: (+46)-8-33 13 99, E-mail: yihai.cao@ki.se

Supplemental Figures

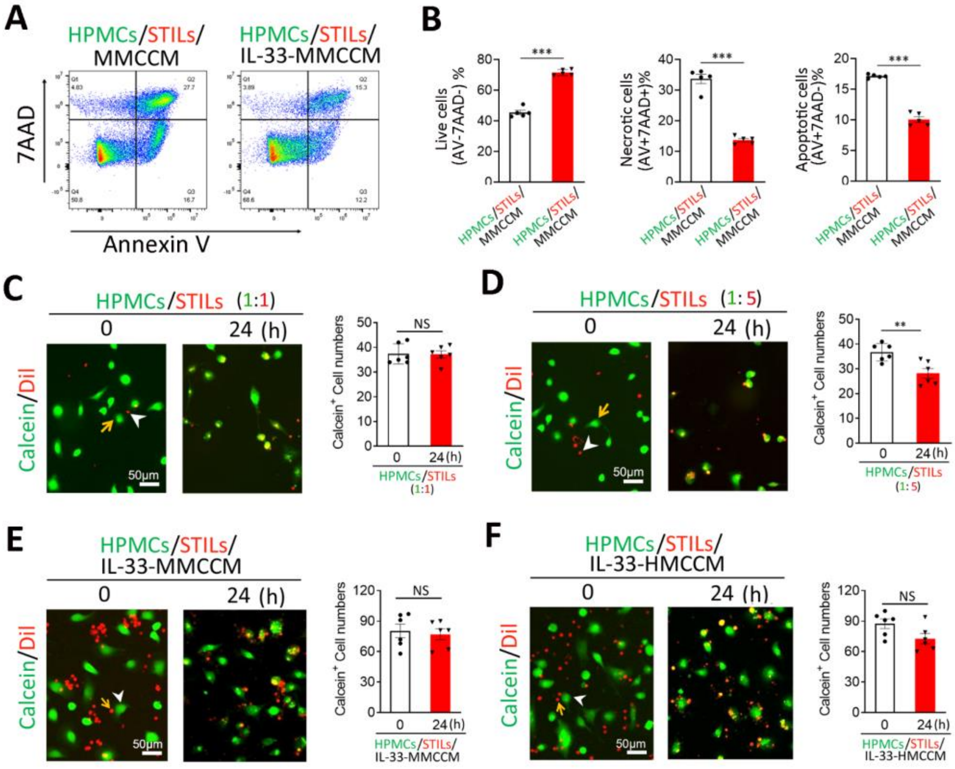

Figure S1. TIL-mediated tumor cell killing and conditioned media from the IL-33-stimulated macrophage display immunosuppression.

A and **B.** FACS analysis to quantitatively measure the proportion of cellular apoptosis and necrosis of tumor cells. Annexin V and 7AAD staining were used to define the necrosis and apoptosis populations in the coculture system. **C** and **D**. TIL-mediated tumor cell killing after 24 h incubation**. C.** At the ratio of 1 : 1, TILs did not display significant killing effects on HPMCs. **D.** At the ratio of 1 : 5, TILs significantly killed HPMCs. **E** and **F.** Randomized micrographs of STILs (red)-HPMCs (green) coculture system with the conditioned media from the IL-33-treated MMCs and HMCs. Yellow arrows point to STILs and white arrowheads indicate HPMCs. **E**. STILs plus HPMCs (10 : 1) cultured with the conditioned media from the IL-33-treated MMCs. **F.** STILs plus HPMCs (10 : 1) cultured with the conditioned media from the IL-33-treated HMCs. Calcein-positive HPMCs in all groups are quantified (n = 6 random fields per group, 10 x magnification). Scale bar = 50 µm. Data are

mean determinants ± SEM; * $P< 0.05$; **$P < 0.01$; ***$P < 0.001$; NS, not significant, Unpaired Student's *t*-test.

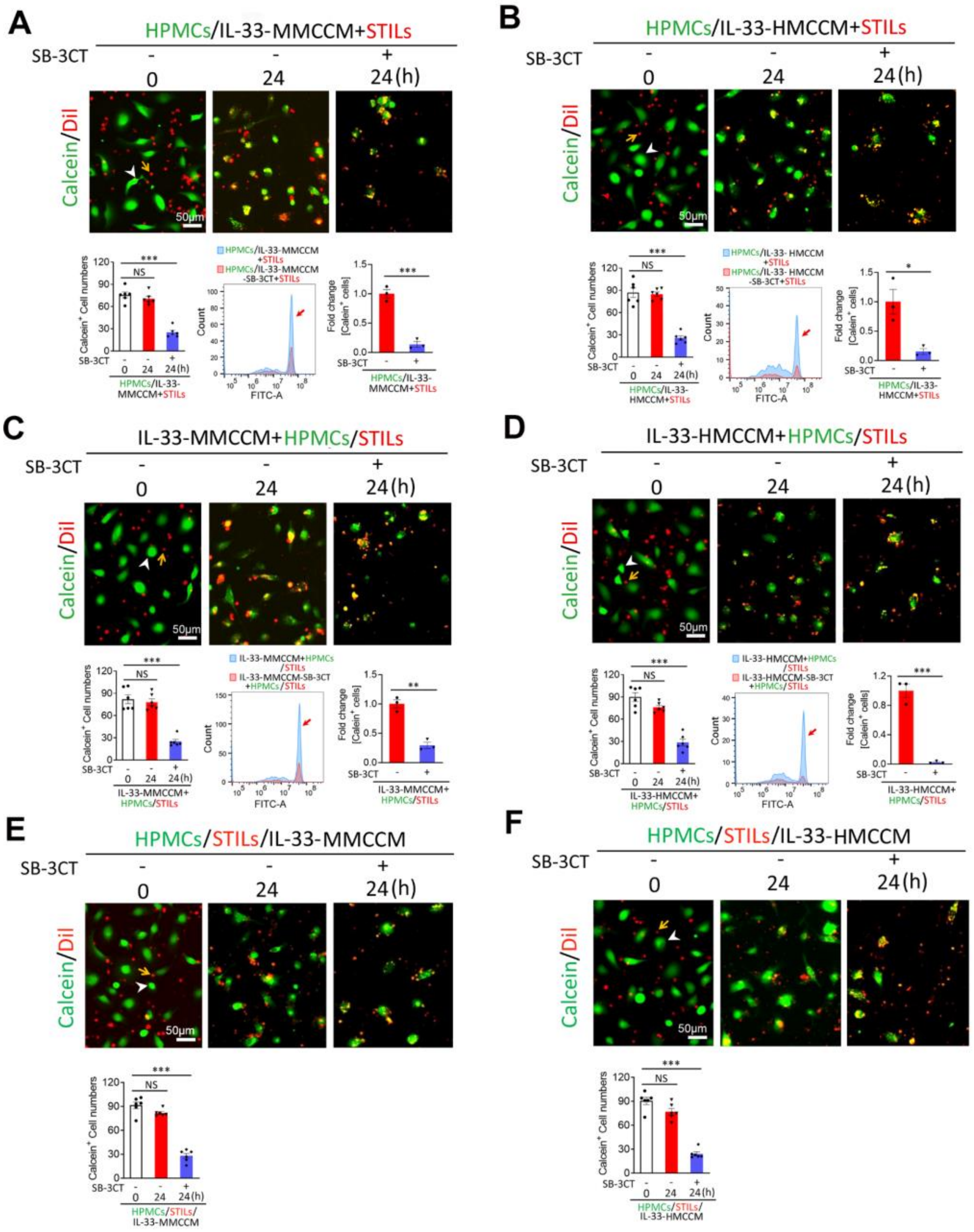

Figure S2. MMP-9 inhibitor restores the IL-33-macrophage-instigated immunosuppression.

A-F. to macrophages. **A.** STILs treated with IL-33-MMCCM with or without SB-3CT plus HPMCs (10 : 1). **B.** STILs treated with IL-33-HMCCM with or without SB-3CT plus HPMCs (10 : 1). **C.** STILs plus HPMCs treated with IL-33-MMCCM with or without SB-3CT (10 : 1). **D.** STILs plus HPMCs treated with IL-33-HMCCM with or without SB-3CT (10 : 1).**E.**

Coculturing STILs and HPMCs in IL-33-MMCCM with or without SB-3CT (10 : 1). **F.** Coculturing STILs and HPMCs in IL-33-HMCCM with or without SB-3CT (10 : 1). Calcein-positive HPMCs in all groups are quantified (n = 6 random fields per group, 10 x magnification). Scale bar = 50 μm. Data are mean determinants ± SEM; n = 6 samples per group. * *P*< 0.05; ***P* < 0.01; ****P* < 0.001; NS, not significant, Unpaired Student's *t*-test.

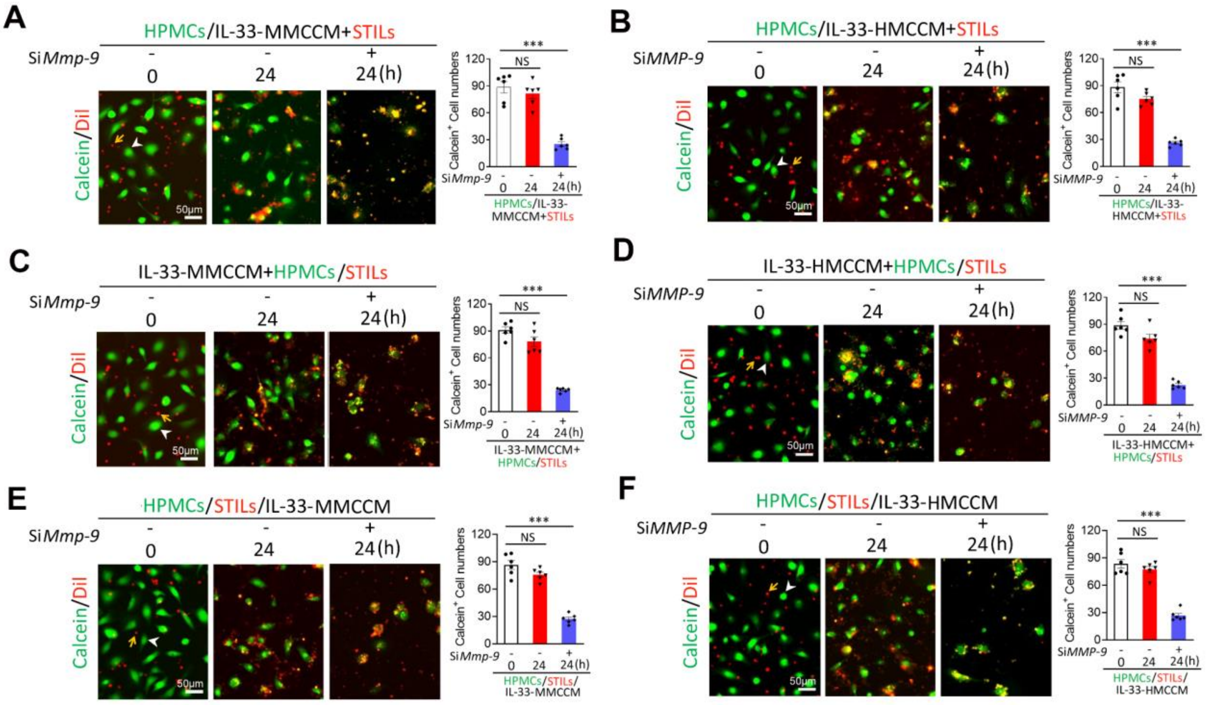

Figure S3. MMP-9 knock-down restores the IL-33-macrophage-instigated immunosuppression.

A-F. to macrophages. **A.** STILs treated with IL-33-MMCCM with or without si*Mmp-9* plus HPMCs (10 : 1). **B.** STILs treated with IL-33-HMCCM with or without si*MMP-9* plus HPMCs (10 : 1). **C.** STILs plus HPMCs treated with IL-33-MMCCM with or without si*Mmp-9* (10 : 1). **D.** STILs plus HPMCs treated with IL-33-HMCCM with or without si*MMP-9* (10 : 1). **E.** Coculturing STILs and HPMCs in IL-33-MMCCM with or without si*Mmp-9* (10 : 1). **F.** Coculturing STILs and HPMCs in IL-33-HMCCM with or without si*MMP-9* (10 : 1). Calcein-positive HPMCs in all groups are quantified (n = 6 random fields per group, 10 x

magnification). Scale bar = 50 µm. Data are mean determinants ± SEM; n = 6 samples per group. * $P< 0.05$; ** $P < 0.01$; *** $P < 0.001$; NS, not significant, Unpaired Student’s *t*-test.

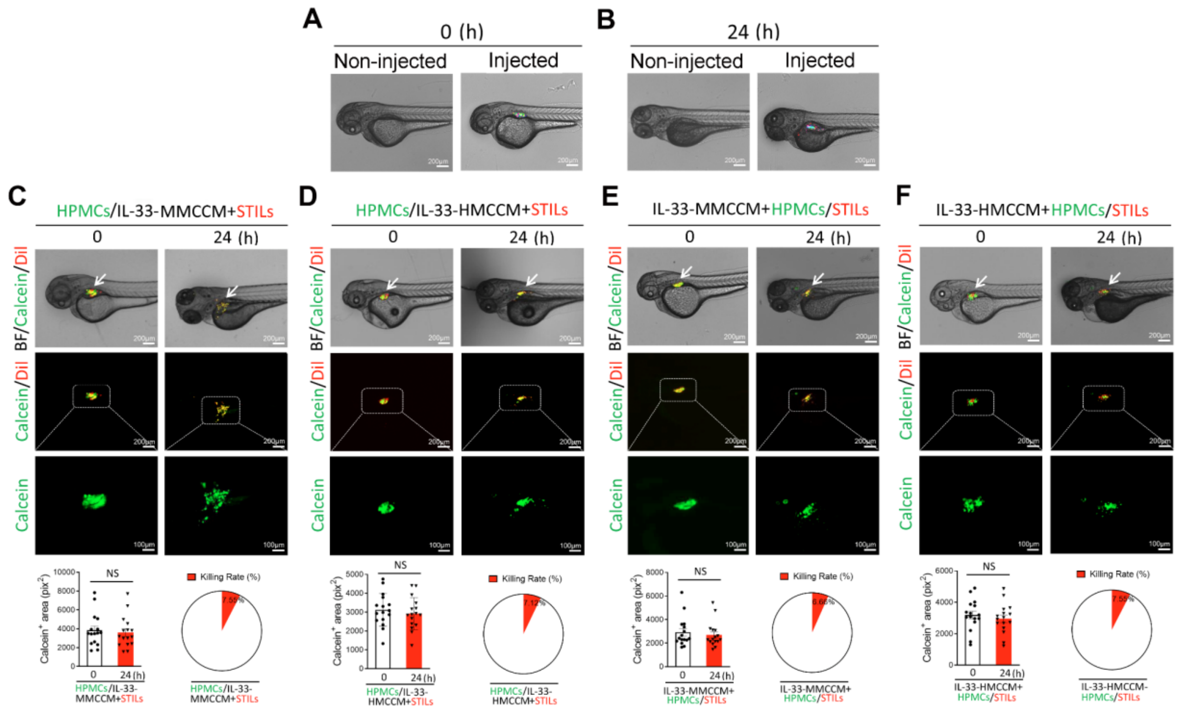

Figure S4. IL-33-stimulated macrophages supernatant prevent HPMC from TILs killing in zebrafish.

A and B. Morphology of zebrafish Implanted with human TILs, HPMCs, and HMCs at 0 h and 24 h. Non-implanted zebrafish were severed as controls. Representative micrographs of calcein-labeled HPMCs (green) plus DiI-labeled TILs (red) were collected at 0 h and 24 h after co-implantation into the zebrafish. **C-F,** White arrows point to injected cells. **C**. STILs treated with IL-33-MMCCM for 24 h plus HPMCs (5 : 1). **D.** STILs treated with IL-33-HMCCM for 24 h plus HPMCs (5 : 1).**E.** Coculturing STILs and HPMCs treated with IL-33-MMCCM for 24 h (5 : 1). **F.** Coculturing STILs and HPMCs treated with IL-33-HMCCM for 24 h (5 : 1). Dashed lines rectangular and amplify the indicated regions. (Scale bars: 200 µm; amplified fields, 100 µm). Quantification of calcein-positive areas in the zebrafish and killing rates of TILs were calculated (a, n = 16 samples per group; b, n = 16 samples per group; c, n

= 16 samples per group; d, n = 16 samples per group). Data are mean determinants ± SEM; * $P< 0.05$; ** $P < 0.01$; *** $P < 0.001$; NS, not significant, Unpaired Student's *t*-test.

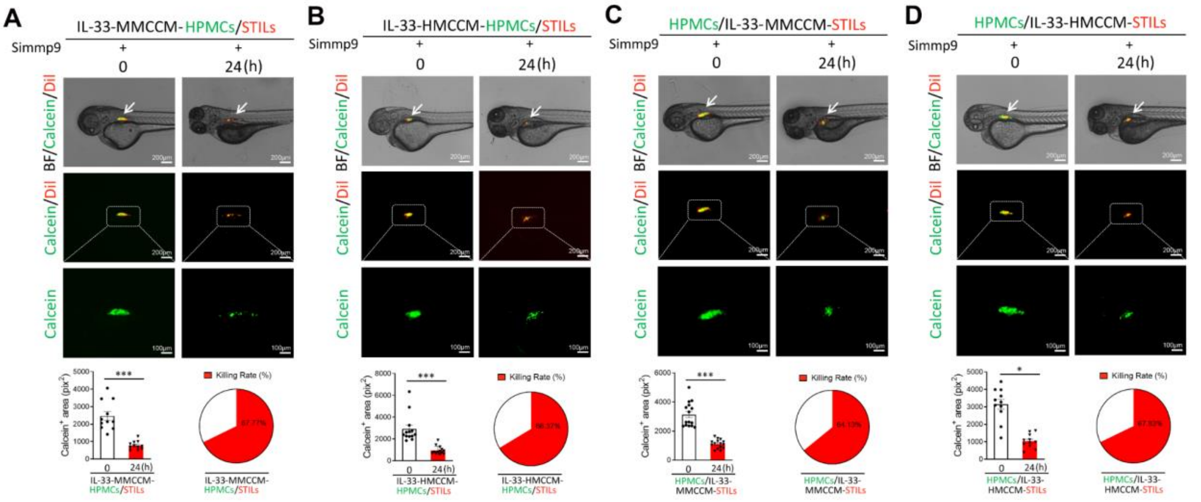

Figure S5. MMP-9 inhibition restores TIL-mediated cancer killing effect in zebrafish. Micrographs of zebrafish bearing HPMCs (green), IL-33-stimulated maacrophages (blue) plus STILs (red) at 0 h and 24 h post-implantation. **A-D,** White arrows point to injected cells. **A.** STILs treated with IL-33-MMCCM with si*Mmp*-9 plus HPMCs (5 : 1). **B.** STILs treated with IL-33-HMCCM with si*MMP*-9 plus HPMCs (5 : 1). **C.** STILs plus HPMCs treated with IL-33-MMCCM with si*Mmp*-9 (5 : 1). **D.** STILs plus HPMCs treated with IL-33-HMCCM with si*MMP*-9 (5 : 1). At 0 h and 24 h post-implantation, quantification of calcein-positive areas in the zebrafish and killing rates of TILs were calculated (a, n = 11 samples per group; b, n = 13samples per group; c, n = 15 samples per group; d, n = 11 samples per group). Data are mean determinants ± SEM; * $P< 0.05$; ** $P < 0.01$; *** $P < 0.001$; NS, not significant, Unpaired Student's *t*-test.

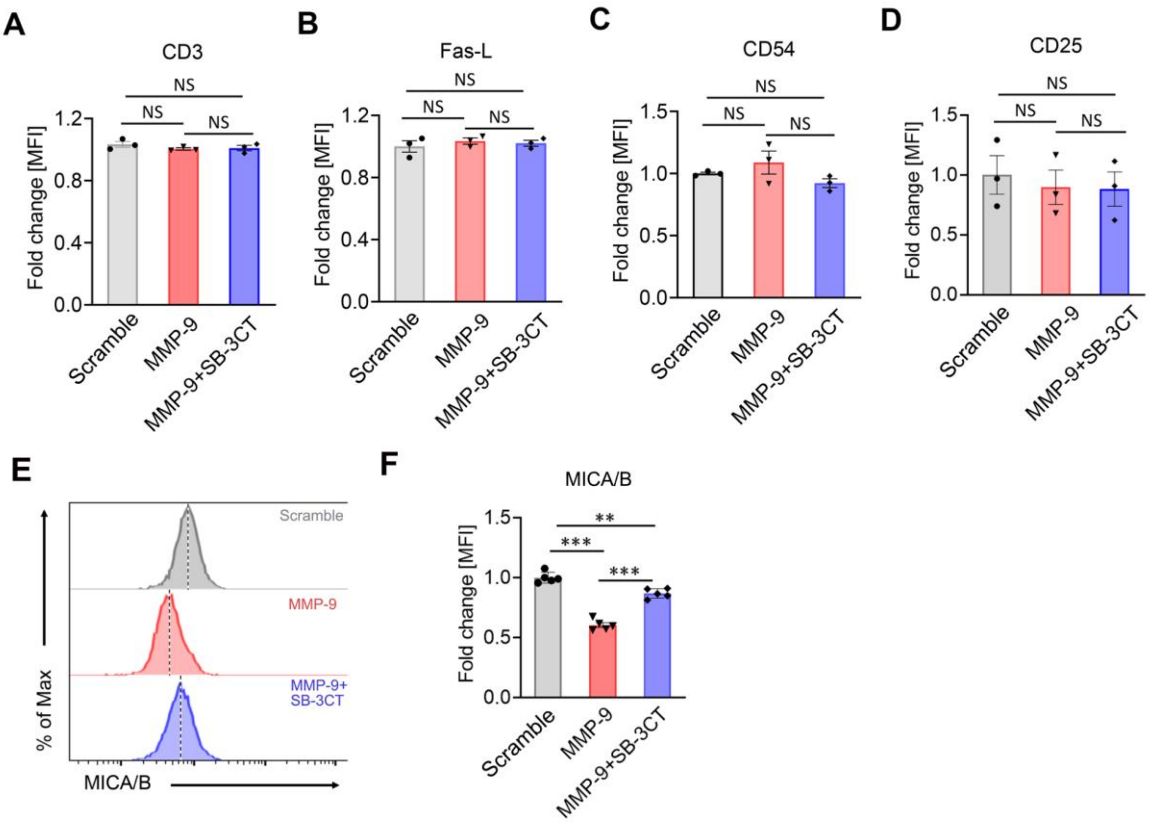

Figure S6. MMP-9 failed in shedding other cell surface markers in TILs.

Purified STILs were stimulated with rhMMP-9 (5 μg/mL) in the presence or absence of SB-3CT (20 μM) for 24 h. **A-D**. The expressions of CD3, Fas-L, CD54 and CD25 on STILs were analyzed using FACS (n = 3 samples per group). **E-F.** The effects of MMP-9 and SB-3CT on cell surface expression of MICA/B (n = 5 samples per group). Data are mean determinants ± SEM; * *P*< 0.05; ***P* < 0.01; ****P* < 0.001; NS, not significant, Unpaired Student's *t*-test.

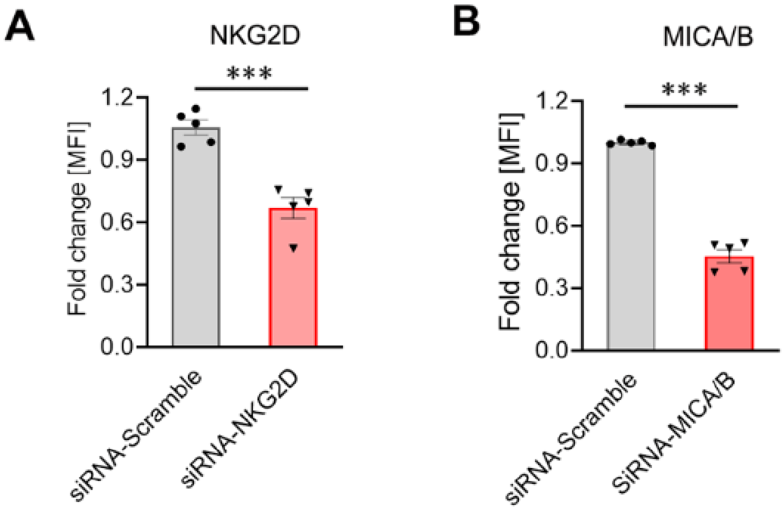

Figure S7. Knockdown efficiency of NKG2D in TILs and MICA/B in HPMCs by specific siRNAs.

A. NKG2D expression levels were analyzed in control scrambled- and NKG2D-transfected TILs by FACS (n = 5 samples per group). **B.** MICA/B expression levels were analyzed in control scrambled- and a mixture of MICA/B -transfected HPMCs by FACS (n = 5 samples per group). Data are mean determinants ± SEM; * *P*< 0.05; ***P* < 0.01; ****P* < 0.001; NS, not significant, Unpaired Student's *t*-test.
